# From technique to normativity: the influence of Kant on Georges Canguilhem’s philosophy of life

**DOI:** 10.1007/s40656-023-00573-8

**Published:** 2023-04-06

**Authors:** Emiliano Sfara

**Affiliations:** Institute of Biology; National Institute in Science and Technology in Interdisciplinary and Transdisciplinary Studies in Ecology and Evolution (INCT-IN-TREE), Federal University of Bahia, Bahia, Brazil

**Keywords:** Canguilhem, Kant, Technique, Critique of Judgment, Philosophy of life

## Abstract

Many historical studies tend to underline two central Kantian themes frequently emerging in Georges Canguilhem’s works: (1) a conception of activity, primarily stemming from the *Critique of Pure Reason*, as a mental and abstract synthesis of judgment; and (2) a notion of organism, inspired by the *Critique of Judgment*, as an integral totality of parts. Canguilhem was particularly faithful to the first theme from the 1920s to the first half of the 1930s, whereas the second theme became important in the early 1940s. With this article, I will attempt to show that a third important theme of *technique* arose in the second half of the 30s also in the wake of Kant’s philosophy, especially Sect. 43 of the *Critique of Judgment*. This section, which states that technical ability is distinguished from a theoretical faculty, led Canguilhem to a more concrete and practical conception of activity. I will then suggest that it was by considering technique that the concept of normativity, which characterizes Georges Canguilhem’s philosophy of life, also took shape.

## Introduction

Three classic figures of philosophical thought exerted great influence on the evolution of Georges Canguilhem’s ideas. Indeed, in an explanatory footnote of the fourth volume of Canguilhem’s *Œuvres Complètes*, Camille Limoges (2015, p. 65) observes that “Auguste Comte […], with Descartes and Kant, is one of the authors associated with every stage of Canguilhem’s philosophical reflection”. I intend to analyse the impact of only one, Immanuel Kant, and I will do so with particular reference to Canguilhem’s philosophy of technique^1^ and life. At the same time, I will look at Kant to understand how a certain interpretation of technical act in Descartes and Alain^2^ was interpreted by Canguilhem.

In philosophy, as in other disciplines, being influenced by an author means to critically engage with their ideas because they have been received through the teaching of a Master. Being influenced can also mean being inadvertently conditioned by an author’s ideas while developing our own philosophical concept. Inversely, we may willingly choose to take on a specific concept from a past author without fully agreeing with them. In such a case, we might say that this author has conveyed a certain influence on us, inasmuch as they have led the choice of some of our ideas.

As per Canguilhem, the influence of Kant was decisive: when commenting on it, some Canguilhem scholars, like Yves Schwartz (2011, p. 80, 81), alluded to “the special space afforded to Kantianism in the philosophical heritage of the young Canguilhem”, while acknowledging that “Kant certainly does not disappear from his later teaching.” Massimo Marianetti (1994, p. 78) went further, saying: “Canguilhem’s philosophical project [was] a fruitful extension of that transcendental conception that stretches from Kant to the present day.” This influence has not gone unnoticed, and there are now several authors seeking to uncover the analogies that link Kant to a “decidedly Kantian historian of science like Canguilhem” (Sánchez Madrid, 2017, p. 138). If the conclusions advanced by these authors make the manifold influence of the Prussian philosopher clear, so do Canguilhem’s own writings, which frequently refer to many of Kant’s works, from the celebrated *Critique of Pure* Reason (Canguilhem, 2015, pp. 361–63) to the *Attempt to Introduce the Concept of Negative Magnitudes into Philosophy* (Canguilhem, 2018, p. 525), and from the *Conflict of the Faculties* (Canguilhem, 2019, p. 776) to the *Anthropology from a Pragmatic Point of View* (Canguilhem, 2015, p. 711).

Despite this ubiquity, however, it has been pointed out that certain elements of Kantian philosophy had a particularly strong influence on Canguilhem^3^ at different times: Xavier Roth (2010, 2011, 2013) has shown that there is an argument, borrowed mainly from the *Critique of Pure Reason*, which had a particularly strong effect on the early phase of Canguilhem’s writings. According to this argument, which appeared to fade in later years, activity is the abstract process through which “the mind orders and regulates a diversity of impressions” (Roth, 2010, p. 4^4^).

It is known that, according to Kant, the intellect—that is to say, judgment—is an active force that moves towards things in the world so as to order them coherently and thus generate knowledge.^5^ Roth argues that the young Canguilhem was clearly impressed by this conceptualisation of activity as a mental and abstract operation of the intellect, but also that this changed in approximately 1937, the year that he published the article “Descartes et la technique.” From that moment—which roughly coincided with the start of his doctoral studies in medicine (1936), with his decision to distance himself from the pacifism of Alain^6^, and with the start of a political involvement that eventually led him to join the French Resistance—Canguilhem’s support for the philosophy of the synthetic act of judgment became “more discreet” (Roth, 2011, p. 614). While retaining some of the important aspects of this old conceptualisation (which indeed will not be completely abandoned and will remain in his further texts as well), Canguilhem gradually moved towards a philosophy of life that he would discuss fully in the famous *Essai sur quelques problèmes concernant le normal et le pathologique*, published in 1943.

In the works written by Canguilhem in the early 1940s, as well as in those of his later years, references abound to another keystone of Kant’s literary production, the *Critique of Judgment*. Several studies (Marianetti, 1994; Debru, 2018; Gayon & Petit, 2018; Limoges, 2018) have called attention to the impact of this work on Canguilhem. For instance, *La connaissance de la vie* (1952), which Canguilhem “placed in continuity with his thesis of medicine of 1943” (Limoges, 2018, p. 27), is strongly imprinted with an “organisational” conception of life that clearly echoes Kant (Gayon & Petit, 2018, p. 130). According to this idea, which Kant formulated in the third *Critique*, “every part of a living being is the cause of the production and the maintenance of the other parts” (Gayon & Petit, 2018, pp. 130–131). Although I shall offer more evidence further on, we need only read an exchange of opinions between Canguilhem and François Dagognet about what most characterises the living being (“self-formation,” Canguilhem exclaimed to a shocked Dagognet)^7^ in order to perceive the decisive theoretical significance that this view of the organism^8^ as a *totality* of interdependent parts assumed in his reflections.

An analysis of the primary and secondary sources therefore demonstrates that two Kantian themes— that of activity as a mental and abstract synthesis of judgment, mainly inspired by the first *Critique*, and that of the organism as a solidary totality of parts, taken from the third *Critique*—recur frequently in two broad but distinct phases of Canguilhem’s work. Canguilhem was particularly faithful to the first theme from the 1920s to the first half of the 1930s, while the second theme fully emerges in the unpublished pages of a university course entitled *Organisme et totalité*^9^ and largely devoted to the subject in 1941.

In between, from 1937 to 1940, there is a kind of transitional phase in which, according to Roth (2011, p. 615), “a philosophy of judgment moves little by little towards a decisive encounter with the philosophy of life.” This, of course, begs the question of what happened to the teachings of Kant during this intermediate period, which was marked by Canguilhem’s departure from Alain’s pacifism, by an imminent war demanding an urgent response, and by three texts^10^ centred on the concept of technique.^11^ Was Canguilhem still under Kant’s influence or had it diminished? One thing is certain: as he reduced his references to abstract judgment (from 1937 onwards), Canguilhem argued instead that the processes of knowledge are triggered by concrete practice.

According to this idea, which as we will see is inspired by a specific interpretation of Descartes, the non-material activity of the intellect cedes its cognitive primacy to practical action. If the theme of technique emerges in all its force during these “transitional” years, it is because technique contains a purely manual “power” (Canguilhem, 2011, p. 490)—the ability to construct an instrument—and allows activity to freeing itself from the judgment. According to Roth, however, the origin of Canguilhem’s concept of technique, which implies the irreducibility of practice to theory, is not to be found in Kant’s philosophy. Roth has the merit of underlining that Canguilhem was prominently influenced by Kant in his conception of synthetic and abstract activities, especially until the first half of the 1930s. Nonetheless, Roth does not mention Kant when highlighting how the irreducibility of concrete action to abstract theory (i.e. technical activity) became a fundamental idea in Canguilhem’s work after the first half of the 1930s.

In this article, I intend to demonstrate, contrarily to Roth, that the new primacy that Canguilhem accorded to concrete action from the mid-1930s can also be traced back to the philosophy of Immanuel Kant, and specifically to the Kant who in the third *Critique* (despite what had been partly established in the first) asserted the distinction between theory and practice.

Michele Cammelli (2022) suggests that Canguilhem – who was originally influenced by the first *Critique* and by the concept of judgment as an abstract activity – later adopted the concept of practical activity as separated from the abstract one, as presented in the third *Critique*. In this respect, Cammelli writes that “a remarkable gap between the Kant of the third and the first *Critique*” appeared in Canguilhem (Cammelli, 2022, p. 57). This provoked a true “short-circuit” (Cammelli, 2022, p. 59) or, better, a *U-turn* in Canguilhem’s philosophy. Even though Canguilhem did not completely abandon the philosophy of judgment, he began to conceive the activity as something that was no longer abstract but, on the contrary, merely concrete. In this article, drawing from Cammelli’s intuition, I will claim that the theses for which the practical act is distinct from abstract theory can be found in a specific section of the *Critique of the Power of Judgment*: § 43 (Kant, 2000, p. 183 – KU, AA 05: 303–304. 29 − 03), which Canguilhem mentioned four times in his published and unpublished writings.

The section, in which Kant claims that practical ability is “distinguished from science (to be able from to know) as a practical faculty is distinguished from a theoretical one, as technique is distinguished from theory,” is in my opinion one of the elements of Kant’s opus that most inspired the philosophy of technique and life developed by Canguilhem.

In order to demonstrate the influence of § 43 on Canguilhem’s philosophy of life, I will focus the next two sections of this paper (Sects. 2 and 3) on a chronological analysis of Canguilhem’s texts in such a way as to face the reader with the abovementioned U-turn in Canguilhem’s conception of activity. In fact, in Canguilhem’s early writings – which I will analyse in Sect. 2 and that lasted more or less from the end of the 1920s to the first half of the 1930s (see Canguilhem, 1924, 1929-32, 1931-32, 2011, p. 319) – activity is mainly conceived as a mental and abstract process that synthetises an irregular flow of external data, in accordance with the Kantian and transcendental philosophy adopted by Alain and other neo-Kantian philosophers (such as Lachelier, Lagneau, Brunschvicth, and Boutroux). After the 1937’s article on Descartes, which I will present in Sect. 3, activity is conceived by Canguilhem as the merely concrete process, absolutely detached from abstract thought, through which an object or a tool is manufactured (Canguilhem, 2011, p. 490–754). According to Canguilhem, this concrete process is called *technique*, and its first conceptualisation can be found in Descartes’s philosophy. Indeed, by betraying his renowned mechanism and rationalism for which a practical act always springs from an abstract thought, in some of his works Descartes claimed that the manufacturing of an object is independent from abstract thought. In Sect. 4, then, I will discuss § 43 of Kant’s third *Critique*, which asserts that practical and technical acts are independent from abstract theory. While emphasising a remarkably close connection between Kant and Descartes, § 43 also reveal a strong divergence from Cartesian mechanism and classical rationalism. This aspect was explicitly underlined by Canguilhem himself when referring to § 43 in a text from 1962 to 1963. In Sects. 5 and 6, I will explain how Canguilhem might have been influenced by § 43 while moving towards a conception of activity based on the irreducibility of practice to abstract theory. In particular, in Sect. 5 I will observe that, around the mid-1930s, Canguilhem (who, by no accident, started his doctoral dissertation in medicine right at that time) was taking distance not only from Alain’s pacifism, but also from the theoretical positions of his teacher – including those on medicine. Alain, just like Descartes in his traditional reception, had a mechanistic concept of the human body. For him, a living being was a machine that obeys to physical-mathematical laws and whose structure explains and commands practical moves within an environment. As I will show in Sect. 6, Canguilhem criticised this conception in a text from 1947 to 1948, in which § 43 is once again explicitly quoted to argue the opposite: that practical movement cannot be guided by abstract rules. Nonetheless, I will also show that there are two contrasting theoretical concepts in Alain’s work. On the one hand, a mechanistic concept of the living being that is in line with his interpretation of the Kantian transcendental judgement. On the other hand, a concept of technical activity that favours a distinction between practice and abstract theory. According to Canguilhem, the latter concept is in accordance with § 43 of the third *Critique.* Nonetheless, Alain was also guilty of not having emphasised it enough in Descartes’ works, even though he sometimes privileged the distinction between practice and theory already underlined by Kant. My claim is that Canguilhem, in a theoretical controversy with his teacher, decided to underline himself this conception of technical activity in “Descartes et la technique” (1937). In doing so, I claim, he might have been inspired by § 43. In Sect. 7, I will then remark that the conception of practical activity thus conceived was fundamental for a subsequent development of Canguilhem’s philosophy of life – once again, in contrast with Alain’s theory of the living being. In his text on the normal and the pathological (1943), Canguilhem argues that a body can change its physiological norms when its real-life activity changes in a given context. This means that it is not the physiological constant that (mechanically) determines the concrete behaviours of a living being. Rather, it is the variation in the recurrence of a given concrete movement that influences the frequency of some physiological constants in a given environment. This is what normativity means, according to Canguilhem. In my opinion, if we define (as it has been done) Canguilhem as a philosopher of the concept, we should not simply understand the term “concept” in its abstract sense, but rather in the technical and concrete sense of a practice conducted in a dynamic and historically variable environment. Finally, in the conclusions, I will assess the theoretical relevance of the present article against recent interpretations of Canguilhem’s work. I will explain in what extent my thesis agrees with and differs from these interpretations. Moreover, my account of Canguilhem’s philosophy of life will lead me to draw some analogies with recent developments in philosophy of biology. Indeed, Canguilhem’s philosophy of life conceives the living being as an entity exerting a concrete influence on its environment. Similarly, some recent philosophers of biology partly inspired by Kant (Moreno & Mossio, 2015; Walsh, 2015; Desmond & Huneman, 2020) propose a reading of the organism from the point of view of its agency, that is, of its ability to concretely operate in a certain context.

In summary, the main argument of this paper is that Canguilhem distanced himself from Alain’s mental and abstract concept of activity, which was borrowed from the first Kantian *Critique*, to end up with a practical and concrete conception of activity in line with § 43 of the third *Critique*. I have no intention to deny the evident heterogeneity of the texts composing Canguilhem’s works, which differ in themes and interests insofar as they are connected to various chronological and biographical stages of Canguilhem’s life. Nonetheless, by adopting this point of view, I will offer a reading of his writings that emphasises a “common thread” running through all of them: the theme of practice. Of course, I am not claiming here that, in the first *Critique*, Kant did not determine the fundamental importance of the practical dimension as far as reason was concerned, nor that he began to do so only in the third *Critique*. It is too well-known that, in the first *Critique*, Kant stated that pure reason should “draw back within the boundaries of its proper territory, namely practical principles” (Kant, 1998, p. 671 – KrV, A794/B822). It is even more widely known that the practical dimension is so important for the second *Critique* to be quoted already in the title: *Critique of Practical Reason.* Rather, I will argue that the theoretical move from the first to the third *Critique* (as well as the shift in Canguilhem’s conception of activity it entails) is specific to the theoretical and biographical journey of Canguilhem and not necessarily faithful to the theoretical evolution of Kant’s concept of practice. My claim is that his early texts on technique and artistic creation, i.e. on the concrete realization of an object, probably led Canguilhem to a close-reading or a strong assimilation of the theoretical concepts of § 43 (focused precisely on practice as technique or art, as we will see later on). Consequently, when alluding to Kantian ideas – like the one regarding the abstract activity of thought – I will be referring to a specific interpretation of Kant by Canguilhem’s teachers (or Canguilhem himself), rather than to a strict exegesis of Kant’s philosophy. Therefore, it is not my intention to discuss very complex questions that are intrinsic to an extremely debated and still unsolved, such as the correct interpretation of Kant’s work strictly based on Kant’s texts or on the Kantian scholarship^12^.

However, this due caution does not prevent me from providing a personal interpretation of why Canguilhem, from the second half of the 1930s, was influenced by the concept of practice presented in § 43 of the third *Critique*, rather than by that from the first and the second *Critique*. Indeed, in the first *Critique* Kant argues that reason is a “practical faculty in itself” (Kant, 1998, p. 454 – KrV, B425), and that “the greatest and perhaps only utility of all philosophy of pure reason […] has only the silent merit of guarding against errors” (Kant, 1998, p. 672 – KrV, A795/B823) – the errors we encounter while looking for the truth, during practice. The reason is, therefore, practical in so far as it has the task of guiding practice in order to prevent practical errors. On the contrary, from 1937 onward, Canguilhem suggests that error, among other things, springs from the technical-practical movement (Talcott, 2019), which may run into error only because it remains at the mercy of chance, that is, because it is detached from reason. Practical error is thus necessary for reason, as abstract reason is compelled to elaborate on the error in order to prevent it in the future. Accordingly, reason is not a practical faculty in itself, since practice is separated from the abstract-theoretical level to which reason belongs.

It is worth noticing, here, that the distinction between the abstract level (theory and science) and the practical-technical level (art) is set by Kant precisely in § 43 of the third *Critique.* In other texts and particularly in the first two critiques, Kant tends not to establish the distinction of reason and practice as presented in § 43. Rather, he links his concept of practice to the metaphysical idea of freedom (“everything is practical that is possible through freedom”, Kant, 1998, p. 674 – KrV, A800/B828), to practice understood as a moral conduct (see e.g. the second *Critique*) or as a national or international law (such as in the text of 1793, see Kant, 1974), and to other more or less similar meanings that are rather distant from that of practice as in § 43. Before elaborating on this point, let me address in detail how Canguilhem interpreted the first *Critique* prior to the second half of the 1930s.

## The young Canguilhem and the first *Critique*

I began by asserting that, according to Limoges (2015, p. 65), the three authors present in every era of Canguilhem’s reflection were Kant, Comte and Descartes. Roth agrees with this assessment, but adds an interesting nuance when he states that by the late 1930s, “Canguilhem’s philosophical pantheon […] *apart from Alain and Lagneau*, was essentially composed of Descartes, Kant and Comte” (Roth, 2011, p. 614, my italics). The reference to the first two figures is not insignificant, since the Kantianism that so impressed the young Canguilhem can be traced back to Alain and, as has also been observed by Schwartz (2011), to Jules Lagneau.^13^ Indeed, Roth (2010, p. 4) continues, “until the mid-1930s, Canguilhem remained loyal to the […] reflexive philosophy whose main representatives are Jules Lachelier,^14^ Jules Lagneau, and Alain, Canguilhem’s teacher.” Just as Canguilhem was a pupil of Alain (at the *Lycée Henri IV* in Paris), Alain had studied under Lagneau, whose university lecturer, in turn, had been Lachelier. Canguilhem himself recalled that Alain had “a certain direct knowledge of important philosophies like Cartesianism and Kantianism passed on to him by personages of the calibre of Lachelier or Jules Lagneau, who was Alain’s teacher” (Canguilhem, 2018, p. 330).

As for the meaning of this reflexive philosophy and the extent to which it was connected to Kant, we must turn to Roth (2010, 2011, 2013), who has devoted various works to this subject. He explains that it is “Lachelier [who] lays the foundations of the reflexive style in philosophy. According to him, Kant inaugurated a new philosophical method.” This method induces “thought to be directed not onto objects […] but onto itself.” In short, thought acquires importance because “its activity is reflected onto [the objects]” (Roth, 2013, p. 82), or, as Jacques Piquemal (1985, p. 66) wrote, simply because it brings to light the “omnipresent activity of judgment.” Of course, the term *judgment* should be construed in Kantian terms, and Roth (2013, pp. 82–83) in fact emphasises that “the *Critique* [*of Pure Reason*] had demonstrated that knowledge derives its value not from things but from the activity of the mind […]. In effect the problem that Lachelier sets himself […] is not so different to that of Kant […]. Lagneau, Alain and the young Canguilhem were keenly aware of this.”

Now, by virtue of the direct filiation between these philosophers and as a consequence of the interpretation of the intellect as an *act*, it may be said that Lachelier, Lagneau, Alain and Canguilhem essentially formed a “school of activity”: it is not a coincidence that Roth named one of his works *Georges Canguilhem et l’École française de l’activité* (2010). According to this “school” (Roth, 2010, p. 231), judgment—ergo thought—does nothing more than confer a coherent order on a chaotic continuum of empirical impressions, on a disordered flow of external data that it perceives and brings to a synthetic unity. The result of this process is called “synthesis” (Roth, 2010, p. 72). Kant, in the first *Critique*, states that the knowledge of different perceptible objects arises precisely from this *active* process of thought, which leads to the unity of experience, to the transcendental synthesis:

By synthesis […] I understand the action of putting different representations together with each other and comprehending their manifoldness in one cognition (Kant, 1998, p. 210 – KrV, A77/B103);

The manifold of representations […] is an action of the understanding, which we would to designate with the general title *synthesis* in order at the same time to draw attention to the fact that we can represent nothing as combined in the object without having previously combined it ourselves, and that among all representations *combination* is the only one that is not given through objects but can be executed only by the subject it-self, since it is an act of its self-activity. (Kant, 1998, p. 245 – KrV, B129)

To use once more the words of Roth (2010, p. 72), “Kant profoundly altered the way of conceiving knowledge. With him, knowledge ceases to be interpreted as a passive contemplation of ideas and is converted into activities of synthesis.” What is worth underscoring is that, according to the young Canguilhem and his “Kantian teachers” (Roth, 2010, p. 232), activity, in keeping with Kant’s teaching, is linked principally to judgment, to thought, to intellect: it identifies an abstract procedure.

In summary, neo-Kantianism had a salient influence on the young Canguilhem through his “Kantian teachers” of that time (Roth, 2010; Schwartz, 2011). Some scholars, such as Fedi (2018) and Beaufret (1984), already provided a number of analyses on their reflexive style and their debt to Kant. Herein referring to these works for a more detailed account, let me just outline some general concepts from these “Kantian teachers” in order to give a better idea of what we are discussing. For instance, in *Du fondement de l’induction* (his most renowned work), Lachelier called “induction” a mental operation that actively converts the heterogeneity of the empirical world – just like the Kantian transcendental synthesis. In fact, in Lachelier’s opinion (1896, p. 37), “Kant introduced into philosophy” the principle according to which, through induction, thought imposes an order on external phenomena. As per Jules Lagneau, he is renowned for his studies on the phenomenon of perception, among other things. Similarly to Lachelier with induction, and thus to Kant with the transcendental synthesis, Lagneau conceived perception not as a passive reception of external data imprinted on the eye, but rather as an active movement of the spirit (*esprit*) that gathers together various sensations from the extended world. “Sensation is in itself a state of the sensing subject. But sensation would not exist in this subject if it were not at the same time active, if the subject did not possess the power of joining together, by the unity of its action, the various sensations”, Lagneau wrote. And he added: “This action, which is properly perception, consists in the determination of the represented qualities as linked to one another in the extended world” (Lagneau, 1964, pp. 265–266). At the same time, we should not forget that one of the philosophers that Canguilhem held, as he explicitly stated,^15^ in “the highest regard” during his youth was Léon Brunschvicg (1869–1944), who was also widely inspired by Kantianism (see e.g. Fedi, 2001). It is not by accident that he was Canguilhem’s teacher at the École Normale Supérieure. Brunschvicg is also indicated by some scholars (Roth, 2010, p. 325; Fedi 2018, pp. 618–619) as a representative of the reflexive style, alongside Lachelier, Lagneau and Alain. For Brunschvicg, in fact, everything a subject sees results from a mental activity that puts in order the discontinuous flow of external sensations. Human vision takes shape, according to Brunschvicg (1954, p. 73), “as the activity of mind becomes broader and more harmonious. We need an activity of ordering our sensations […] to get at a glance the whole of objects which are arranged in our terrestrial horizon”. In addition, it is worth remembering that Brunschvicg wrote the preface to the French translation of *Des vérités éternelles chez Descartes*^16^ by Émile Boutroux (1845–1921), a neo-Kantian French philosopher who was also theoretically close to the positions of the French reflexive style (see Roth, 2010, p. 234; Schwartz, 2011, p. 80). Incidentally, the French translation of this work was edited by the young Canguilhem, who was considerably influenced by Boutroux (Guillin, 2008, pp. 66–67).

But let us take, now, the case of Alain, who, as the Kantian that he was, affirmed that “Kant is the head of the school, and blessed be he. Blessed be the desert of Kant. It is here that the probity of the spirit is learned, and that it is learned again and again” (Alain, 1936, p. 190). A high school teacher and author of numerous works of philosophy, including *Propos sur le bonheur* (1925), *Vingt leçons sur les Beaux-Arts* (1931) and *Idées* (1932), Alain was Canguilhem’s teacher at the *Lycée Henri IV* in Paris in the first half of the 1920s. Appealing to the transcendental demands of the “illustrious Kant” (Alain, 1900, p. 8), he conceived of thought as an abstract action that, by applying a *synthesis* to a multiplicity of heterogenous data, represents it as a coherent unity. In “Le problème de la perception,” Alain writes for example that “on a coloured surface, even a small one, there is a multitude of different shades […]. But the indefinite and the indeterminate cannot be grasped as such. Thus, what we believed to be a simple and primitive sensation, actually results from the application of unity to multiplicity, that is, from the very action of thought” (Alain, 1900, p.10). In the same way, reiterating that “the action of thought not only makes things contiguous, but constitutes the things themselves in the way that we perceive them” (Alain, 1896, p. 623; quoted by Roth, 2010, p. 80), he wonders, “is this power to make one what is multiple and finite what is infinite not what we call thought? Is it therefore thought that makes movement possible?” (Alain, 1893, p. 530; quoted by Roth, 2010, p. 79). To Alain’s mind, the answer can only be positive. Thought is in fact the repository of a “law that unites all my possible sensations” (Alain, 1902, p. 8), that is, an abstract principle of synthetic unity. In the *Lettres à Sergio Solmi sur la philosophie de Kant*, he ends up declaring that “the unity of *I think* is the first of the metaphysical objects, it precedes all the others. It precedes God, even in simple consciousnesses; before believing in God, you have to believe in yourself” (Alain, 1946, p. 24). Canguilhem was certainly convinced by his master’s teachings, so much so that, referring to his younger self, he described himself as a follower of “Alainism” (Bing & Braunstein, 2018, p. 1292).

This Alainism emerges early on in a series of notes taken by Canguilhem when attending a course on the philosophy of Kant delivered by Alain in 1924. What is immediately apparent from these notes is that Canguilhem had fully assimilated the Kantian idea, inherited from his teacher, of an intellect (*entendement*) construed as the “power to lead the heterogeneity of intuition to the unity of perception” (Canguilhem, 1924, p. 10). And since perception is supported by intellect, as Alain had demonstrated in his “Le problème de la perception,” for Canguilhem it is “much more of an action than a contemplation” (Canguilhem, 1931-32, p. 59), as he would later write in his notes for a series of lectures that he gave as a high school teacher.

At this point, we are still far from the mature Canguilhem (1991, p. 34), who was intrigued by the “concrete human problems” brought up by his study of medicine and biology. In the pre-1937 Canguilhem, the influence of Kant, via Alain, is not so much in issues linked to practice or to concrete action. Instead, this influence concerns the transcendental value (abstract, linked to the intellect) that Canguilhem attributes to a whole series of philosophical ideas, concepts and fields of knowledge. In a manuscript written between 1929 and 1932, for example, he states that psychology, far from being “the study of biological individuality,” is “a reflexive analysis of the permanent and universal conditions of every thought or, to use the words of Kant, a transcendental critique” (Canguilhem, 1929–32, p. 167). Elsewhere, we read that work (*travail*), in contrast to the common understanding, has nothing to do with practical or manual activity, since it “is essentially the intellect in action” (Canguilhem, 1931-32, p. 17). The conception of activity that emerges repeatedly in the writings of the young Canguilhem is that of a mental activity, conceived as a synthesising operation of abstract and transcendental judgment.

On the other hand, his admiration for Alain led Canguilhem to support his teacher’s committed pacifism and to write numerous articles of a political or philosophical nature “in Alain’s journal, the *Libres propos*,^17^ of which in 1932 he became editorial secretary” (Braunstein, 2000, p. 11). In one of these, published on 20 January 1930, Canguilhem (2011, p. 269) uses numerous quotes from the first *Critique* to respond to those^18^ who blamed Kant for insufficient rationalism: “in my opinion, the solution offered by Kant is not at all insufficient and […] rationalism could not have done any better.” He also explicitly invokes Kant in a brief article published in *Europe* only a few months later: “my thought, such as it is, is subject to an absolute rule of unity (what Kant calls the originally synthetic unity of apperception)” (Canguilhem, 2011, p. 319).

The first *Critique’*s hold over the young Canguilhem was so unyielding that, if we look at the definitions that he sometimes gave to certain key philosophical concepts, we cannot fail to see that these can be traced directly back to ideas borrowed from Kantian philosophy. If “judgment is the essential act of thought” (Canguilhem, 1929–32, p. 166), the real condition of the possibility of experience is provided by the “schema,” as Kant indicated in the first *Critique*: “the schema is not produced either by pure intellect or by pure feeling, but by an intermediary power that Kant calls imagination, whose function is to produce movement. The scheme is a possible image-building process.” Subjects act only after considering the images suggested by the schema, and it is for this reason that “the schema is the condition of possibility of experience” (Canguilhem, 1929–32, p. 163). If the transcendental subject represents, through images, a succession of phenomena linked to each other by a rule, in other words by a principle of unity that is the condition of possibility, it is possible because of the schema.

The young Canguilhem (1929–32, p. 163) goes on to state that it is also on the basis of what Kant^19^ calls a schema or “schematism of our understanding” that “the perception of the noticeable multiplicity” takes place “through the unity of the intellect.” Here, then, we reach the heart of reflexive philosophy, of which Canguilhem, by way of Alain, was a “direct descendent” (Roth, 2010, p. 78), at least until the mid-1930s. If in Alain (1900, p. 753) activity was a process well suited to thought, since “it results from the application of unity to multiplicity,” the same conceptualisation can be found in Canguilhem, according to whom the “production of movement” schema absorbs the multiplicity of the outside world into the unity of thought.

## The question of technique

From the mid-1930s onwards, Canguilhem’s conception of activity as a mere mental and abstract process appears to have gone through a seemingly radical theoretical reversal. While it is true, as Roth (2010, p. 219) rightly points out and as we shall see shortly, that even “from 1935 to 1939 the problem [borrowed from the first *Critique*] of the unity of experience is clearly at the heart of his [Canguilhem’s] reflections,” it is also the case that in the same period the concept of activity often points to a different action, one that is practical, material and free from the bonds of the abstract and transcendental intellect.

The irreducibility of practical action to the abstract plane of the intellect is consequently at variance with the Kantian theme of judgment: the primacy of action that emerges from Canguilhem’s work from the second half of the 1930s is opposed, on this point, to the theses put forward by the abovementioned reflexive philosophers. As Roth claims, if Canguilhem “is interested in the problem of technique it is because it constitutes, in his eyes, an original experience whose intelligibility escapes […] the reflexive paradigm of the masters.” This, in turn, lead him to “highlight the irreducibility of action with respect to the judgment of knowledge” (Roth, 2010, p. 220).

The reason for this theoretical U-turn is to be found in the four important writings that Canguilhem added to his body of work in that period, having hitherto mainly produced brief philosophical-political discussions or newspaper articles. The writings were: *Le fascisme et le paysans* (1935), “Descartes et la technique” (1937), “Activité technique et création” (1938) and the *Traité de logique et de morale* (1939, co-authored with Camille Planet^20^). On the one hand, there is no doubt that “the theories put forward by them are rooted in a philosophy of justice” (Roth, 2010, p. 219) and consequently in the arguments first set out in the *Critique of Pure Reason* that refer to the unity of experience and to the establishment of a philosophy as an “integral experience” 21 (Canguilhem, 2011, p. 500). To give an example, being nothing more than “a professor of unity”^22^ (Canguilhem, 2011, p. 501), the philosopher must teach that the subject merely synthesises his own empirical-social experience in a judgment or a set of judgments that in turn become authentic values, that is, ideals or behavioural objectives, such as the value of truth, which “alone can and must guide judgment” (Canguilhem, 2011, p. 800). Even so, for the first time in Canguilhem’s published work and at variance with the Kantian theme of judgment, these four texts rely strongly on the subject of the irreducibility of practical action to the mental and abstract plane of the intellect. This is especially true for the last three. To be clearer, the concept of practical action to which I refer is presented in the introduction to “Descartes et la technique.” This concept is identified with what Canguilhem defines as *technique*^23^ or, indeed, *practice*. In that introduction, the author sets out to interrogate Cartesian philosophy by means of the following question:

Is technical activity a mere extension of objective knowledge […] or is it the expression of an original “power,” creative in its essence, and for which science would elaborate, from time to time afterward, an agenda or a code of precautionary measures? Cartesian philosophy seems to have approached this important problem head on and to have considered the relationship between theory and practice in a broader and more nuanced way than is generally assumed. (Canguilhem, 2011, p. 490)

Let us suppose that one wanted to make a clock for the first time. Would it be enough to follow an instruction manual in order to be sure of obtaining the desired outcome? Classic, traditional Cartesianism would say that it is, and in fact it is well known that Descartes believed that to manufacture an instrument or a tool it is necessary to adhere to a series of preliminary rules produced by the intellect that will guide and orient the practical-technical procedure at all times.

Moreover, it is this idea that underpins the celebrated Cartesian rationalism, as emerges very clearly, for example, in the twenty-one *Rules of the Direction of the Mind*. Canguilhem was well aware that “from the first rule on, Descartes opposes the diversity of technical attitudes […] with the unity of theoretical intelligence and aims to arrive at it through integral knowledge” (Canguilhem, 2011, p. 494): consequently, the philosopher of the *cogito* “scorns art without explanation (I, 195)^24^ and inventors without method (X, 380), being extremely distrustful of artisans who do not work under the direction of the rules he suggests.” Insofar as the sum of these intellectual rules constitutes a *science*, Descartes does not look kindly on those who, claiming to be able to manufacture tools by allowing themselves to be guided by pure chance, do not understand the physical sciences, as is made clear by rule V (Descartes, 1985, pp. 20–21).

Against the “traditional” Descartes, prince of the intellect and pioneer of rationalism, Canguilhem brings to light a kind of little-known “overturned” Descartes who postulates the chronological antecedence of practical (technical) action over theory, over the rules of the intellect. The 1937 article cites certain examples drawn from various parts of Descartes’ works in support of this fundamental thesis: Descartes recommends a balanced approach to practical-empirical efforts that carefully avoids starting out from a series of precautions and theoretical rules, since otherwise “theory would be ridiculed by practice (II, 469).” Similarly, five years after having elaborated the laws of the telescope, Descartes points out to Mersenne, regarding the construction of this instrument, that there is a difference between theory and practice because, Canguilhem (2011, pp. 495–496) continues, in the *Dioptrique* it is written that the telescope “was first found by experience and luck” (Descartes, 1963, p. 651).

By the same token, this is why “knowledge of nature depends doubly, from the *Dioptrique* onwards, on human technique: in the sense in which the instrument […] helps to discover new phenomena (VI 81 and 226), but principally in the sense that technical imperfection provides the opportunity to conduct theoretical research through the difficulties that must be resolved” (Canguilhem, 2011, p. 496). Apropos of this, the article published a year later, “Activité technique et création”, stated that the science of thermodynamics had its origins in the effort to discover why a mechanical device like the steam engine had performed so erratically and disappointingly. Similarly, the theories of Pasteur—including his observations on anomalies in the preservation of wine—were formulated after certain “technical accidents” (Canguilhem, 2011, p. 503). This is confirmed in numerous passages of *Georges Canguilhem and the Problem of Error*, in which Samuel Talcott (2019, p. 86) accounts for Canguilhem’s conception of technique by using a specific expression that can be found in the *Traité*. There, Canguilhem and Planet claim that practical-technical ability is characterized by a series of “trials and errors” (Canguilhem & Planet 2011, p. 685; see Talcott 2019, e.g. p. 86, pp. 90, 100–102) through which a concrete object takes shape during its realisation. The error thus construed is, therefore, creative as such – to the extent that it cannot be thought beforehand, as it precedes the abstract level of the theoretical (and therefore scientific) premeditation. Yet, it is precisely insofar as the “technical experimentation is creative” that “thanks to its very failures, it also creates the possibility of pursuing theoretical knowledge” (Talcott, 2019, p. 82) and allows the formulation of scientific theories, like those by Pasteur.

In short, just as theory comes after practice, science comes after technology. The latter is also a genuinely aesthetic phenomena, not unlike art. If technique falls squarely within a so-called “philosophy of creation,” then art unquestionably corresponds to a “will of integral creation” (Canguilhem, 2011, pp. 502, 503): the artist (sculptor, painter etc.) cannot know the result of the work that he sets out to produce until after he has manually and concretely completed it. He is therefore not guided by theoretical rules, but instead driven by a pure desire to create, by a simple *power*: by a praxis. It is for this very reason that Canguilhem defines technique as something that is found “somewhere on the path of universal manufacture between life and art” (Canguilhem, 2011, p. 505). In parallel to this, the 1939 *Traité*, far from examining the problems linked to logic and ethics in a merely traditional way, contains long passages on the very concept of the chronological and theoretical antecedence of technique and practice with respect to science and theory. For example, in a section entirely devoted to the relationship between technique, art, and science Canguilhem (2011, p. 687) writes that “every time one is faced with a scientific problem it is because a technical failure^25^ […] has aroused feelings of wonder or suffering […]. Without medicine, without the domestication of animals and the cultivation of plants, biology would be nothing.”

In the same way, he once more brings Descarte’s *Dioptrique* into play when he asserts that “the optical theory of lenses presupposes a process of glass manufacturing and treatment, i.e. a pre-existing technical product” (Canguilhem, 2011, p. 666). It is in this particular sense, by borrowing the words *analysis* and *synthesis* from the vocabulary of the first *Critique*,^26^ that a relationship is established between “theoretical analysis and synthetic action” (Canguilhem, 2011, p. 801). In other words, science as analysis and technique as synthesis are related to the extent that “no scientific method can lead to synthesis, but this means once again that life is the first form of this synthetic activity of which technique and art constitute for humanity a more or less pure exercise” (Canguilhem, 2011, p. 754).

## Section 43 of the third *critique*

What, at this point, can be said about Kant’s sway over the Canguilhem of the second half of the 1930s? Did Kant’s thought, like that of the teachers of the reflexive school and in particular Alain, cease to be a source of inspiration for the concept of the irreducibility of practice (technique) with respect to theory (science). Put differently: once the primary dimension of the intellect ceased to be the *conditio sine qua non* of real experience, did Kant’s influence fade away? The first thing that one notices is that in the texts in which the distinction between practical action and abstract intellect is set out—in other words, in the two articles from 1937 and 1938—there is no explicit reference to Kant or his works.

However, if we wished to take a *philological* point of view to interrogate the works of Kant directly, we might ask whether the combination of technique and science, construed in terms of an irreducibility of practice with respect to theory, was actually borrowed from Kant in the same way that the first *Critique* inspired the philosophy of the young Canguilhem through the “dichotomy analysis/synthesis” (Schwartz, 2011, p. 82) or through the concept of *judgment*. There is a passage in the writings of Kant that corresponds to the second part of § 43 of the *Critique of Judgment* (1791), entitled “On art in general” (in the section devoted to the *Critique of Aesthetic Judgment*). This passage seems to refer to the same concept that Canguilhem used to express the idea of the irreducibility of practice (or technique, or art) in comparison to theory or science. It states:

Art as a skill of human beings is also distinguished from science (to be able from to know), as a practical faculty is distinguished from a theoretical one, as technique is distinguished from theory (as the art of surveying is distinguished from geometry). And thus that which one can do as soon as one knows what should be done is not exactly called art. Only that which one does not immediately have the skill to do even if one knows it completely belongs to that extent to art. Camper describes quite precisely how the best shoe must be made, but he certainly was not able to make one. (Kant, 2000, p. 183 – KU, AA 05: 303–304. 29 − 03)

Let us give thought to the final sentence for a moment. Petrus Camper (1722–1789) was a Dutch scientist who, despite writing a textbook on shoemaking, could not make even one shoe.^27^ In this respect, he was not far from the Descartes described in the 1937 article, who, while accepting the essential role of an optical theory of lenses, admitted that the telescope had been discovered thanks “to experience and luck,” that is, thanks to practical movements unconnected to any theory. There seems therefore to be a close analogy, both terminological and conceptual, between the technique described by Canguilhem and the technique described by Kant in § 43. This was in fact made plain by the Italian scholar Massimo Marianetti (disciple of the philosopher Silvestro Marcucci, who had helped spread Kantian philosophy in Italy)^28^ in a 1994 article entitled “Canguilhem, Kant and ‘transcendental philosophy’”: “the problem encountered by Kant is the same as Canguilhem’s. He, as Kant had already done […], connected the reflection on art (technique) to the reflection on science. […] In § 43 of the *Critique of aesthetic judgment*, Kant defined art, or technical ability, as a *power* distinct from the *knowledge* of science, as something not deducible or reducible to it” (Marianetti, 1994, pp. 51–52).

The similarity between § 43 and Canguilhem’s concept of technique, which, as in Kant, is “distinct from science, like power from knowledge and practice from theory,” has also been noted in a more recent article by Fiorenza Lupi (2019, pp. 133–134). As said, however, there is no reference to Kant in Canguilhem’s 1937 and 1938 texts, which dealt exclusively with the subject of science and technique. Accordingly, no affinity with § 43 was explicitly mentioned by Canguilhem in these texts. However, during a seminar from the 1960s given over entirely to these two subjects (the notes of which are contained in a file entitled *Science et technique*),^29^ Canguilhem noted that the person who studied the relationship between science, technique

and art was Kant. *Critique of Judgment*, § 43. “On art in general.” […] Art [as] a human ability distinguishes itself from science as power [*pouvoir*] does from knowledge and technique from theory […]. What one can do [*ce que l’on peut*] as soon as one knows [*dès que l’on sait*] what one has to do is not art (it is science) […] (Example of Camper): “Camper describes quite precisely how the best shoe must be made, but he certainly was not able to make one” […].

The Kantian theory implicitly constitutes an organicist reaction against the mechanism axiomatized by Descartes. (Canguilhem, 1962–63, pp. 10–11)

We shall shortly analyse in more detail the reference to the “mechanism axiomatized by Descartes.” As we shall see, in fact, it is also with reference to Descartes’s philosophy that the mature Canguilhem criticised the intellectualism of his teacher, Alain, that is, his excessive tendency to base philosophical belief on the chronological and theoretical antecedence of intellect rather than on practical action. This was a form of criticism that arose in a specific historical situation.

## The historical-political context and the distancing from Alain’s conception of medicine

A few months before his death, Canguilhem (2018, p. 1292) gave a famous interview in which he talked about an “Alainist” phase of his youth (which started in the mid-1930s) but declared that it had “passed, and what made it pass was, to be specific, the occupation, the resistance and […] medicine.” According to the critical bibliography by Limoges (1994, p. 401), Canguilhem began his doctoral studies in medicine in 1936. A year earlier he had published, in the name of the CVIA (Comité de Vigilance des Intellectuels Anti-fascistes), *Le fascisme et les paysans*. “Canguilhem was the anonymous author of this sixty-two-page document,” Limoges (1994, p. 401) wrote. “The Comité de Vigilance des Intellectuels Anti-fascistes was created in response to the February 1934 riots in Paris and the threat of fascism, and it remained in existence up to the war. […] Canguilhem himself was quite close to the action of the committee. The booklet has three parts,” in which the author “dealt with the consequences of fascist totalitarianism in rural areas.”

We must bear in mind that Alain, although having regularly coordinated the *Comité* alongside Paul Langevin^30^ and Paul Rivet^31^ (Limoges, 1994), took strictly pacificist positions, in line with what had previously always been his political approach. Canguilhem, like his teacher, had been a staunch pacifist right up to the middle of the 1930s (very clearly expressed in the title of an article from 1932, *Peace without reservation? Yes* [Canguilhem, 2011, pp. 400–411]) but changed his position when the Nazis began to knock on the door of rural France. In his text on fascism and the farmers, Canguilhem set out a specific political need that for the first time called for concrete action capable of opposing the “French agrarian movements inspired by fascism,” as defined by Cammelli (2011, p. 518). Convinced of the fact that it was not possible to “come to terms with Hitler,”^32^ Canguilhem abandoned the “old” pacifism of his master and, a few years later, joined the French Resistance that had sprung up in response to the occupation of France by Nazi troops.

However, let us return for a moment to the interview quoted above. What does it mean that Canguilhem distanced himself from Alainism because of “medicine”? How did Alainist thought conceptualise the functioning, internal constants and physiology of the human and animal body? In an article from 1952 discussing the salient features of the philosophy of Alain, Canguilhem had little doubt on this point: “Alain borrows from Descartes the idea that the living body is a machine whose structure explains and commands movement, and not the other way around” (Canguilhem, 2008b, p. 65). “A sick man,” Alain (1940, p. 300) observed, “is a man who no longer controls his physical environment, who no longer governs his machine.” The Alainist cure for illness, therefore, had to be administered in accordance with the mechanical-mathematical principles of Cartesianism, a genuinely “superior medicine” (Alain 1939, p. 85). Here, then, from a purely biological point of view, Alain’s conception of living beings was that of subjects largely under the control of principles, or rather abstract “laws,” that adhered to Cartesian physics and mathematics: “biology, or the science of life, evidently offers greater difficulties, since living beings, whatever their law may be, are inevitably subjected to chemical, physical, astronomical and even mathematical laws” (Alain 1939, p. 192). Hence, it was not surprising that before the mid-1930s the young Canguilhem was aligned not only with the Kantian doctrine of judgment, but also with Alain’s radical pacifism and with the reading of Descartes that he propagated. During this phase, as already pointed out by Guillin (2015, p. 319), Canguilhem’s consideration of the living organism and medicine was affected by the “ascendancy of the Alainist interpretation of Descartes […] to the extent that the insistence on the Cartesian mechanism is precisely one of the specificities of the interpretation in question.”

In a 1930 writing in which he addressed the students of the French upper classes directly, Canguilhem (2011, p. 275), then still a teacher imbued with the belief in the Cartesian mechanism passed down by Alain, warmly recommended an analysis of the fifth part of the *Discours de la méthode*. In it Descartes declared, among other things, that “we do not need to suppose any other cause to impel the most agitated and the most penetrating parts of the blood […] to make their way to the brain rather than anywhere else […] according to the rules of mechanics (which are the same as those of nature)” (Descartes, 2006, p. 45). Nevertheless, the start of Canguilhem’s medical studies marks the moment when he lessened his reliance on “bookish” knowledge (Bing & Braunstein, 2018, p. 1282) and took up a hands-on profession. Medicine provided an “introduction to concrete human problems” (Canguilhem, 1991, p. 34) finally removed from the inconsistency of a series of mechanical-mathematical principles, that is, from abstract rules. Medicine is practised in the clinic, it is a technique, it is the “art of healing,” as Lefève (2014, p. 198) has underlined. The celebrated definition of medicine that Canguilhem (1991, p. 34) offered in his doctoral thesis of 1943 was that “medicine seemed to us and still seems to us like a technique or art at the crossroads of several sciences, rather than, strictly speaking, like one science.”. As scholars have recalled many times (e.g. Roth, 2010; Gayon & Petit, 2018, p. 10), for Canguilhem (1991, p. 33) “philosophy is a reflection for which all unknown material is good”. Thus, it is precisely because it is a field experience that medicine (which is a ‘stranger’ discipline, i.e. unknown to philosophy) is useful for philosophical reflection. According to Canguilhem, two medical techniques (i.e. clinic and therapeutics) “cannot be reduced entirely and simply to a single form of [theoretical] knowledge” (Canguilhem, 1991, p. 34). It is for this reason that they decisively contribute to the formation of philosophical theories that draw upon technical practices such as medicine.^33^

## The theoretical break with Alain: the “biological interpretation” of technique

The irreducibility of practical action to the intellect—a theme of § 43 of the third *Critique*—was, however, certainly not a concept unknown to Alain. Indeed, as an enthusiast of the ideas of Kant, he was at home with the *Critique of Judgment*. In *Histoire de mes pensées*, for instance, Alain (1936, p. 147) declared that he had read it “several times and in the grip of the most intense admiration,” while in *Vingt leçons sur le Beaux-Arts*, he wrote that in order to undertake studies in the Fine Arts it was essential to have read the *Critique of Judgment* (Alain, 1931, p. 6).

This is made even clearer in the above-cited article by Canguilhem which analyses the main elements of Alain’s philosophy: *Réflexions sur la création artistique selon Alain* (1952). While Alain’s philosophy of creation conceived human knowledge as an abstract synthesis of judgment that adhered to the “reflexive” reception of the first *Critique*, on the level of aesthetics—that is, the conception of the genesis of a work of art—he surprisingly affirmed the irreducibility of practice to the abstract plane of the intellect. Human imagination (which for Kant, as we have seen, was closely connected to the intellect as well as to the senses) reaches a point at which it can no longer represent in detail the object of its representation, and it is then that, driven by improvisation, the subject involved in the creation of a work of art attempts to create with his hands what he can no longer imagine. Hence, it is practice that creates works of art, not imagination.

Thus Alain—an intellectualist on the one hand, and an aesthete of the practical act on the other—embodies a certain theoretical ambiguity, a sort of contradiction. “The fundamental paradox of Alain’s aesthetics is that works of art are those things for which only a lack of imagination and the impotence of contemplation […] are responsible,” Canguilhem (2008b, p. 59) states immediately before citing the words of his teacher: “if the power of execution did not extend much beyond the power of thinking or dreaming, there would be no artists” (Alain, 1920, p. 27), and “it is because imagination is incapable of creating […] that the arts exist” (Alain, 1931, p. 108). As Talcott (2019, p. 128) wrote, Canguilhem attributed a central role to Alain on this, since making a portrait implies that “the activity of making comes first and the idea that orders the work emerges only in the course of its making.” Indeed, according to Alain’s aesthetics, “technique and action precede and make possible the object that can be known, but they do so initially without advance knowledge of the object to be known” (Talcott, 2019, p. 128). At this point, however, Canguilhem (2008b, p. 62) stipulates that “this is a Kantian idea. […] When Kant defines doing by distinguishing it from knowing, he insists on the fact that art never involves the application of pre-existing knowledge.” Moreover, immediately afterwards, he specifies that this Kantian idea derives in particular from § 43 of the third *Critique*, which Canguilhem cites in his text. This is mentioned again in an unpublished manuscript from 1947/48 entitled *Le problème de la création*, which also contains references to Descartes who, as we have seen, was another author dear to Alain. Indeed, Michele Cammelli (2022, pp. 55–56) pointed out this double presence in Alain: Kant and Descartes. On the one hand, at the beginning of the foreword to *Système des Beux-Arts*, Alain explicitly stated that his research is dominated by the analyses of Kant’s third *Critique*. On the other hand, he claimed that he was inspired by Descartes’ theory of mechanism, but not by Descartes’ passages on the irreducibility of practice to abstract intellect: “All research on aesthetics […] is dominated by the analyses of Kant’s *Critique of Judgment*, now classic, but too little known in their penetrating details. […] I proceeded differently with regard to a no less important guiding idea, which I found in Descartes, […] defined by mechanism” (Alain, 1920, pp. 11, 12).

There is certainly a sort of twofold “problem” regarding the conception of creation, or the manufacture of instruments in Descartes. On the one hand, there is the traditional Descartes, according to whom creation must gradually follow the rules of the intellect, while on the other hand there is a Descartes in which creation is dictated by practical improvisation. “There are therefore contradictions in Descartes” (Canguilhem, 1947-48, p. 49), contradictions that Canguilhem had rightly noted for the first time in “Descartes et la technique” (1937). However, these contradictions were never spotted by Alain, whom Canguilhem would reproach for exhibiting the same kind of ambiguity as Descartes:

First, the Cartesian theory of the relationship between knowledge and action had to be questioned. This is what Alain did not do. He reintegrates art into technique but only accidentally analyses the postulates of technical activity. Hence, the oscillations in his thought with regard to the nature of technical activity: sometimes he opts for an intellectualist interpretation, and sometimes for a biological interpretation. Intellectualist interpretation: […] with respect to technique as applied to industry, the idea precedes and regulates execution. Biological interpretation: the creation of tools is a special case of the origin of biological species and living forms. (Canguilhem, 1947-48, p. 49)

The point that should be highlighted is that Canguilhem defined as a “biological interpretation” the conception of technique as the irreducibility of practice to theory. Alain, Canguilhem (2008b, p. 63) affirmed, “was able to show that the shape of the fishing boat perfectly adapted to the sea is the result of the progressive reproduction of the best shapes, tested by the environment and by use” and that, likewise, the shape of violins^34^ is the result of a series of practical attempts that depend on the small obstacles encountered during the manufacturing process, in the same way that the shape and physical structure of animals depends on their adaptation to an environment. The type of practical interpretation of technique that can be seen in Alain is therefore biological, since Darwin, the father of modern biology, is one of its primary sources of inspiration (Canguilhem, 2008b, p. 63). At the same time, however, as Canguilhem wrote in the manuscript on the problem of creation, it is inspired by § 43 of the *Critique of Judgment*: “we can liken these reflections of Alain […] to the Kantian idea in the *Critique of Judgment: Critique of Aesthetic Judgment*, § 43” (Canguilhem, 1947-48, p. 49).

## The concept of normativity and the third *critique.* Final remarks on the notion of “concept”

It was with this Kantian but also Darwinian core that the concept of technique elaborated in Canguilhem’s writings from the second half of the 1930s tended to assume an increasingly biological configuration, to such a degree that during the 1940 and 1950 s his subsequent works would become ever more fully committed to an authentic “philosophy of living and life” (Limoges, 2012, p. 65), or to a “biological philosophy” (Gayon, 2006). Indeed, as we have already seen, at the very beginning of his doctoral thesis of 1943, Canguilhem defined medicine as an art or a technique at the crossroads of different sciences, rather than a science in the strict sense. Thus, it is in the wake of the theoretical argument against the “classical” Cartesian conception of Alain, and in perfect harmony with § 43 (with which, however, even Alain’s ideas exhibited signs of continuity), that in these years Canguilhem rejected both the mechanistic conception of the physiology of the living being and a physical-mathematical approach to biology. He (Canguilhem 1947-48, p. 47) explained that “the difficulty of the thesis which constitutes the philosophical mark of Alain’s thought” lies in the fact that “biology is a mechanism,” just as “[classic] Cartesianism” would have it.

It was for this reason that with *The Normal and the Pathological* Canguilhem developed a radically different conception of a living being than that of Alain: “the living creature does not live among laws but among creatures and events which vary these laws. What holds up the bird is the branch and not the laws of elasticity” (Canguilhem, 1991, p. 197). Some interpreters of Canguilhem’s work, such as Méthot (2013, p. 117), insist that the works on technique that we have already discussed merit particular “attention as they are key to understand the origin and the originality of the *Essai sur quelques problèmes concernant le normal et le pathologique*, written in the early 1940s.” This is because the theoretical heart of the concept of technique, previously represented by the precedence of concrete practice over abstract theory, is transmitted as such in the conception of the living that characterised Canguilhem’s work from the early 1940s onwards. If it is true that a human being acts in its own milieu through a technique that is characterized by various practical attempts of “trials and errors” (Talcott, 2019) – which in turn provide a concrete form to tools and artistic objects –, it is equally true that practical error characterises human life in all its concrete activities. “There are technical behaviours, and strictly speaking all vital behaviours are”, Canguilhem remarked (1962-63, p. 7) in an unpublished manuscript entitled “Science et technique”. Similarly, in his work on the normal and the pathological, he claims that “all human technique, including that of life, is set within life” (Canguilhem, 1991, p. 130). Therefore, it can be argued that life itself is an intrinsically technical phenomenon. If science is born from a technical failure, then so is medical science, which originates from an observation of the behavioural and physiological failures a human being experiences in its own environment: diseases.

This means, in a way, that the experience of pathology chronologically precedes physiology. And, as it is well known, this is what Canguilhem made clear on several occasions in *The Normal and the Pathological* (see e.g. Canguilhem, 1991, pp. 178, 203–226). However, it is worth noticing that the argument of the chronological antecedence of pathology over physiology is a theme used, before Canguilhem, by Kant himself, although in a different text from the third *Critique*: *Anthropology* (1798). In the expanded version of the *Essay* (published many years after 1943), Canguilhem stated that he was not aware that this specific topic was present in Kant’s works. Nevertheless, there is no doubt that, similarly to the Kantian-inspired theme of technique (in which the concrete act precedes the moment of theoretical-scientific elaboration), the antecedence of pathology over physiology also implies that concrete experience, in the form of a series of physiological-behavioural failures (diseases), precedes physiological theory. Again, this is a probable Kantian influence coming from § 43. Let me quote Canguilhem:

One young colleague,^35^ a fine Kant specialist studying the Kantian philosophy in its relations with eighteenth-century biology and medicine, pointed out a text to me of the kind that generates at once the satisfaction of a great meeting and the embarrassment at an ignorance under whose shelter one believed one was able to claim for oneself a bit of originality. Kant noted, more than likely around 1798: “[…] It is diseases which have stimulated physiology; and it is not physiology but pathology and clinical practice which gave medicine its start. The reason is that as a matter of fact well-being is not felt, for it is the simple awareness of living, and only its impediment provokes the force of resistance”. 36 (Canguilhem, 1991, pp. 233–234)

As Talcott explains (2019, p. 261), a human being, as such, is “errant”, since the adventure of life places it in front of constant risks from the environment in which it practically operates. Accordingly, medical “science is […] human […] insofar as it is an activity in the service of life’s dangerous ‘trials and errors.’ While Canguilhem uses this phrase only once in the Essay, it clearly draws on his earlier work, and suggests that before error gains its scientific or moral sense, it occurs as a blind straying here and there as the living being attempts to dominate its milieu” (Talcott, 2019, p. 100).

Let us take, for example, one of the central arguments from Canguilhem’s 1943 work, which is based on the concept of “normativity” (Canguilhem, 1991, p. 129). He argues that the body, in certain situations, can change its physiological norms when its real-life activity changes in a particular context. For instance, we might say that an adult human being accustomed to living at low altitudes is in a normal state when its internal physiology manages to adapt to a change in altitude, without experiencing particular ailments. If this does not happen, we can say they are in a pathological state. In an example quoted by Canguilhem (1991, p. 182; see Sfara, 2016, pp. 91-92), a children’s nanny suffers from neuro-vegetative disorder after arriving in the mountains, i.e. when she is suddenly in an environment with atmospheric variables (such as pressure) to which her organism has never or almost never been exposed. As a result, she feels diminished in her ability to perform the daily tasks she usually performed. “Of course, no one is obliged to live at high altitudes”, noted Canguilhem (1991, p. 182), “but one is superior if one can do it, for this can become inevitable at any time. A norm of life is superior to another norm when it includes what the latter permits and what it forbids”. A pathological state, then, arises when a different or unexpected concrete situation surprises the organism, which does not react with a new adaptation norm (which would be the case under normal health conditions).

As we can see, based on normativity, the internal variables of an organism are not regulated in strict accordance with abstract norms, but as norms that vary according to the subject’s practical action in a natural or (as in the case of the human species) social scenario (Méthot & Sholl, 2020). This practical, concrete normativity is the lens through which the physician or biologist correctly interprets the physiology of the body: “this point of view is that of vital normativity. Even for an amoeba, living means preference and exclusion. A digestive tract, sexual organs, constitute an organism’s behavioural norms” (Canguilhem, 1991, p. 136). Moreover, “if it is true that the human body is in one sense a product of social activity, it is not absurd to assume that the constancy of certain traits, revealed by an average, depends on the conscious or unconscious fidelity to certain norms of life. Consequently, in the human species, statistical frequency expresses […] normativity” (Canguilhem, 1991, p. 160). It is therefore not surprising that some Australian aborigines, the Kokatas, have a lower metabolism than other individuals of the same height and weight who live in the United States; or that some indigenous people from South and Central America “have a higher metabolism with a slowed pulse and permanently lowered arterial pressure” (Canguilhem, 1991, p. 174). An individual’s physiological constants are often in line with the average of the physiological constants registered in a given community. If a nomadic population presents some physiological constants different from those that can be measured in a given metropolitan community of Central Europe, it is probably due to everyday practices that differ from the European sedentarism. In the same way, when we look to Africa, « out of 84 Brazzaville natives, 66% showed hypoglycaemia; of these, 39% went from 0.90 g to 0.75 g and 27% were below 0.75 g. […]. From the European point of view, they are pathological” (Canguilhem, 1991, p. 171). However, from the indigenous point of view, these conditions “could almost be considered physiological”, 37 i.e. not at all pathological, since laid down by specific habits of life of the individuals in question. In medicine, we therefore speak of “homo faber” (Canguilhem, 1991, p. 171) inasmuch as the physiological constant is often determined by a set of social practices of an individual that practically (i.e. technically) operates in a specific context.

At this point we are a long way from the Cartesian approach favoured by Alain, which reduced biology to physical, chemical or mechanical laws and likened the physiology of the living body to the functions of the components of a machine. Against this thesis, Canguilhem would repeatedly assert the idea that “biological pathology exists but there is no physical or chemical or mechanical pathology” (Canguilhem, 1991, p. 127), since there is a substantial difference between machines and organisms. Machines are in fact alien to the concept of normativity: the mechanical constancies that regulate their functions are barely conditioned by the external context in which they actually operate. Furthermore, the functioning of a machine, given that it is unaffected by normativity (or adaptation to its milieu) and responds for the most part to a series of physical-mathematical abstract laws, does not enjoy the phenomena of self-regulation that characterise a living organism involved in a real interaction with its context. This theory, which binds the concept of technique to the conception of the organism as an essentially biological phenomenon capable of self-regulating and self-reproducing, was clearly shaped by the third *Critique*, including § 43. Canguilhem himself stated as much in an article published in *Knowledge of Life*, “Machine and Organism” (1952):

Now, contrary to Descartes, one author has affirmed both the irreducibility of the organism to the machine and, symmetrically, the irreducibility of art to science. This is Kant, in the Critique of Judgment […]. In paragraph 65 of the “Critique of the Teleological Power of Judgment” Kant […] distinguish[es] machine from organism. […] But in paragraph 43 (from the “Critique of the Aesthetic Power of Judgment”), Kant defines the originality of this intentional human technique relative to knowledge in an important text. (Canguilhem, 2008, pp. 92–93)

This “important text” is obviously § 43, whose words Canguilhem uses directly in his piece. As for § 65 of the “Critique of the Teleological Power of Judgment”, Canguilhem frequently stressed its importance, even in his private correspondence. In a letter dating 19 January 1993 and addressed to Marianetti,^38^ he wrote that “the paragraphs 64, 65 and 66 of the *Kritik der Urteilskraft* [Critique of Judgement] are in no way called into question by what I have called the normativity of life.”

Another allusion to these three sections (the analysis of which has been the focus of endless literature, including most recently Zumbach, 1984; McLaughlin, 1990; Zammito, 1992; Huneman, 2008; Goy & Watkins, 2014) is also present in the above-cited 1952 article, “Machine and Organism”: “In an organism—and this is too well known to need insisting—one observes phenomena of self-construction, self-conservation, self-regulation, and self-repair. In a machine, its construction is foreign and presupposes the ingenuity of the mechanic; conservation demands the constant surveillance and vigilance of the machinist” (Canguilhem, 2008, p. 88). It is within these sections that Kant (2000, pp. 243–244 – KU, AA 05, pp. 371–372) refers to the “self-help of nature in the case of injury,” and to an organism that “unceasingly produces itself, and likewise […] continuously preserves itself.”

However, the important role of these ideas in the thought of Canguilhem could already be discerned in a course held in 1941, a part of which was devoted to the notion of “purpose according to Kant” (Debru, 2018, p. 302). The organism, which in contrast to the machine is not built by an external entity, is distinguished by its concomitant and unified constituent parts, which generate and regulate each other reciprocally from within: “the organism therefore has a creative property, it is the cause of itself and of its parts. Therein lies the difference with the machine” (Canguilhem, 1941-42, p. 17). An organism’s *raison d’être* is therefore not external (in other words, decided by a builder or engineer), but internal: it has, in the terms adopted by Kant, a “natural purpose” (Canguilhem, 2019, p. 723), an intrinsic one. On this subject, *The Normal and the Pathological* states that “in this sense the Kantian concept of finality is always relevant” (Canguilhem, 1991, pp. 217–218). It is a concept that, pointing to a “totality” of parts that it self-produces and develops by virtue of its practical activities in an environment, “put the total organism and its behaviour […] into the forefront.”

Even after the 1950s, Canguilhem’s writings continued to make use of the Kantian notion of the organism as a self-reproducing totality. For instance, in *Études d’histoire et de philosophie des sciences*, published in 1968, we read that “a machine, says Kant, is a whole in which the parts exist for each other but not because of each other. No parts are built from the others, none are built from the whole […]. A machine does not contain a formative energy.” In the same work there is also a reference to the fact that, starting from the *Critique of the Power of Judgment*, “organisms are totalities whose analytical breakdown and causal explanation are subject to the use of an idea of purpose, which guides all research in the biological field” (Canguilhem, 2019, p. 701; 438). Limoges (2018, p. 28), moreover, comments that when Canguilhem let his “biological philosophy” be guided by certain “regulating ideas, such as those relating to the character of totality or normativity proper to every living being,” he did so “in accordance with the lesson of the *Critique of the Power of Judgment*.” This illustrates the considerable philosophical significance that the Kantian notion of organism assumed in Canguilhem’s later reflections on philosophy of life.

Canguilhem’s philosophy of life, grounded on a Kantian inspired conception of technical-practical act, is directly linked to themes and notions that, at a first sight, may seem incoherent with an organist philosophy of life. Indeed, this is the case of the notion of “concept” – Michel Foucault (1991, p. 8), after all, defined Canguilhem as a philosopher of the concept. However, Canguilhem’s “concept” is not simply an abstract component of human or scientific thought. It doesn’t spring from a simple passive observation of a phenomenon. As underlined by Schmidgen (2014, pp. 238–240) with particular reference to *La formation du concept de réflexe*, for Canguilhem, the concept is also what directs action from within when it comes to manufacturing technical tools that may help science to better understand a phenomenon. The concept is embedded in the technical action of a human being, thus also in that of a scientist that operates in their own historical, social, and environmental context. A concept does not derive from a scientific rationality, but rather comes from a need, a wish, a specific willing of a living being (e.g. the thirst for discovery) and is often expressed in a gross and problematic way that opens up to further clarifications. For instance, the problems inherent to the elaboration of the 19th century concept of reflex brought about – fifty years later – the adjustment of laboratory tools aimed at clarifying the nature of the reflex action (Canguilhem, 1955, p. 161). Consequently, the specificity of tools varies from one historical-social context to the other. However, what remains constant beyond various specific elaborations is the persistence of the concept of reflex, i.e. a certain “conceptual filiation” (Canguilhem, 1955, p. 5) between authors from different centuries and geographical areas, “from Willis and Astruc to Unzer and Prochaska” (Schmidgen, 2014, p. 250). And this because the non-rational need that comes with a concept is inherent to a biological organism (the human being) that throughout the centuries responds in a technical and normative way to their constant needs. Indeed, Canguilhem (1962-63, p. 7) claims that it is from the everlasting human will to find effective remedies to disease that the study of human body and the elaboration of different concepts of reflex were originated. This is why I cannot agree with Marina Brilman (2018, p. 29) when she puts the notion of Canguilhem’s concept – as rooted in the technical experience – at odds with Kant. The latter was not only a philosopher who, with the transcendental judgment, “sought to explain how reason can exist prior to experience and nevertheless correspond to it” (Brilman, 2018, p. 28), but also the one who showed, by the means of § 43 of *Critique of Judgment*, that technical experience is quite distant from reason.^39^ Canguilhem knew how to develop this gap, mainly by defining – in his article from 1937 – the chronological antecedence of technical experience to scientific rationality, as well as by broadening the range of this philosophy of technique so that to encompass an important part of his philosophy of science and life.

## Conclusions

If his philosophy of life was therefore openly influenced by Sects. 64, 65 and 66 of the third *Critique*, Canguilhem pointed out the close similarity between § 43 and the concept of technique, elaborated in his own writings of the second half of the 1930s, only in the *après coup*. This acknowledgment first appears in an unpublished manuscript from 1947 to 48, and then in two texts from 1952 (on artistic creation in Alain and on the difference between the machine and the organism) and in another manuscript that can be dated to the early 1960s (concerning the question of technology and its relationship with science).

We therefore do not know whether Canguilhem was directly inspired by the section in question while drafting the writings from 1937 to 1939. Some handwritten notes that he took on the French version of the third *Critique*, in the margin of § 43, certainly date back to a subsequent period.^40^ This is an important point, but not yet completely clear. It may lead to further research on this topic. For instance, to specific analyses of the unpublished manuscripts written between 1937 and 1939 aimed at detecting a clear influence of § 43. Thus, far from being a definitive study, the present paper could open up a line of research that deals not only with Kant’s transcendental synthesis (Roth, 2010, 2013) or his philosophy of organism (Debru, 2018), but also with Kant’s philosophy of technical act in order to clarify the way it has influenced Canguilhem’s ideas. What does not seem to be in much doubt, in fact, is that Canguilhem had been introduced to some of the main themes of the third *Critique* from a young age, most likely by Alain. Indeed, many of the extracts, notes and explicative passages, in a 186-page notebook dating back to the 1920s entitled simply *Kant*,^41^ relate to the third *Critique*. “The Critique of Judgment,” Canguilhem wrote in these pages, “is at the starting point of romantic philosophy. This means that Kant is not the cold theoretician of the intellect and the severe moralist we often talk about” (Canguilhem, undated, p. 85). On page 85, however, we also read that “in the *Critique of Judgment* we are in the presence of miracles of freedom, works of art […], which reveal themselves as spontaneous productions of nature, close relatives of the manifestations of organic life” (Canguilhem, undated, p. 85).

I believe that behind Limoges’s (2018, p. 48) compelling observation that Canguilhem’s philosophy “imposes itself entirely as a philosophy of action” there is a Kant who is not only convinced that activity is an operation of judgment (as put forward by Alain and his predecessors), but also that activity is a concrete practice of a subject operating in a certain context. This judgment is in accordance with the title of an article by Salomon-Bayet, “Georges Canguilhem, le concept et l’action” (1996), as well as with Méthot’s claim that “the formation and rectification of concepts in Canguilhem’s sense are intrinsically bound with the experimental, material, technical, and cultural contexts in which concepts are operationalized” (Méthot, 2013, p. 112).

In fact, it is the current context—by which I mean the philosophical debate on certain issues that have arisen within recent research in biology—that has provided the impetus for this article. The theme of practical action is still important in the contemporary philosophical debate on biology. To my mind, it is reasonable to discern analogies between the concept of “agency” as formulated by Walsh (2015), Moreno and Mossio (2015), and Desmond & Huneman (2020), and Canguilhem’s notion of normativity. In very broad terms, according to the concept of agency, “organisms are agents with goals and purposes that interact with their environments, and their behaviour can […] be understood with reference to the goals of organisms as wholes rather than as mere collections of parts” (Desmond & Huneman, 2020, p. 34). Contemporary philosophy of biology places increasing emphasis on the actions of organisms and on how they transform their environment, as a transformed environment can have a decisive influence on the evolution of species. Since it seems inappropriate to attribute an action only to single body parts, scholars look at organism as a whole.

Those that defend this conception of agency of an organism conceived as a totality that self-regulates through its concrete interaction with the environment, make reference, not unintentionally, to the conception of organism presented by Kant in the third *Critique*. For this reason, Walsh (2015, p. 10) writes that “unlike machines, an organism’s parts are the consequence of the activities of the organism as a whole. The constituent parts and processes of a living thing are thus related to the organism as a whole by a kind of ‘reciprocal causation’. […] Kant’s own definition of an organism emphasises the reciprocity between part and whole.” Furthermore, Moreno and Mossio (2015, pp. XXIII–IV) point out that “Kant was the first author who defended the view that organisms are deeply different from machines because their parts and activities are non-separable.” This, as underlined by Bateson (2005) and Nicholson (2014), marks a “return of the whole organism” that has sometimes taken place in recent studies on the philosophy of biology. This is the reason why Jonathan Sholl (2020, pp. 258, 259) has recently stated that it was not a coincidence that “Canguilhem’s organismic perspective”, which puts the total organism and its behaviour to the forefront, finds “resonance in later developments in biology and what some call a possible ‘return of the organism’”. Canguilhem was one of the pioneers of this “return”, not only to the extent that Kant was the philosopher of the “whole organism,” but also because, as we read in *L’action*,^42^ “the philosophical concept of the autonomy of action dates back to Kant, since it is with him that the decisive break with respect to the traditional conception of the relationship between acting and knowing takes place” (Canguilhem, 2018, p. 244).

Similarly to what Roth (2010, 2013), Méthot (2013), Limoges (2018), Talcott (2019) and Cammelli (2022) have argued, and in developing some past research (Sfara, 2016, 2018), in this paper I tried to demonstrate how central the theme of the irreducibility of practical activity to thought is in Canguilhem’s philosophy. Roth was able to show how important was Kant’s transcendental philosophy before the theme of the practical-technical action became relevant in Canguilhem’s work. My intention was to indicate how central Kant’s *Critique of Judgment* was for the elaboration of the same topic. In fact, Cammelli (2022) sensed the importance of the third *Critique* with regard to the theme in question, as I have recalled previously (see Sect. 1). Talcott (2019) as well, on his part, sensed that “implicitly, drawing on Kant”, Canguilhem sets “error’s rightful place in human experience and activity”. The aim of my article was to make even more explicit Kant’s influence on Canguilhem’s conception of practical activity as separated from thought through a precise genealogy that originates in § 43 of the third *Critique*.

In conclusion, it is worth underlining the differences between the present research and my previous publications. In some pages of my work from 2018, I claimed that the attention Canguilhem paid to the topic of practical action (as separated from thought) is not limited only to the texts from the second half of the 1930s: if we read some unpublished manuscripts, we can detect this issue, even though occasionally, already in the texts from the 1920s. Canguilhem (1929-32, pp. 195, 209), inspired by the aesthetic of his master Alain (as we showed before, see Sect. 6), wrote for example that “just as judgement can only arise from judgement, action can only arise from action. Action is characterised by original realisations that it neither imitates nor copies (from judgement)” (see Sfara, 2018, pp. 105–106). In a similar manner, but also referring to published texts, Talcott (2019) emphasised the presence (and the importance) of the topic of concrete action already in 1929. For instance, following the lead of the physician and psychoanalyst René Allendy (1889–1942), Canguilhem (1929) hinted at the importance of the concrete interaction with the sick (who has a specific and original personality that the medical practice must consider) in order to obtain a correct interpretation of pathology (Talcott, 2019, pp. 43–49). Nevertheless, while not intending to contradict the conclusions I reached in 2018, with this article I have also indicated the specific point in which the theme of practical action ceases to be a minor one in Canguilhem’s work. Indeed, fuelled by Canguilhem’s rising theoretical-political contraposition with Alain (see Sects. 5, 6), this theme becomes particularly frequent around 1937, before providing a decisive theoretical imprint to many subsequent writings. If this topic – as I have argued in the past (Sfara, 2018) – does not emerge in one fell swoop like Athena from Zeus’ forehead, it might be equally true that it derives from § 43 of Kant’s *Critique of Judgment*, as I have tried to argue in these pages.

## Footnotes

1 *Technique* is a French term Canguilhem uses to denote the whole range of ways of proceeding that characterise a living being (as a human being) when it operates practically in a vital context, e.g., while manufacturing an object. For the purpose of this paper, I will use the English “technique” with this same meaning. Firstly, because the English term also indicates a number of practical activities (even though, semantically speaking, it does not entirely correspond to the French *technique*). Secondly, because the English “technique” has already been used in some translations of Canguilhem’s works, as *The Normal and the Pathological* (see Canguilhem, 1991) and adopted by Anglophone Canguilhem’s scholars, such as Méthot (2013) and Talcott (2019). Nevertheless, let me point out that, from a Canguilhemian perspective, translating the French *technique* with “technology” is not in the least incorrect. Méthot (2013, p. 6), for example, often prefers this translation.

2 Alain was the pen name of Émile-Auguste Chartier (1868–1951).

3 On Canguilhem’s evolving views on Kant, see (Rand, 2011; Brilman, 2018; Etxeberria & Wolfe, 2018).

4 In this paper, I will mainly refer to Roth’s PhD thesis (2010) and not to the partially amended version published in 2013. During an interview dated 18th of October 2019, Roth told me that in order to comply with the editorial guidelines of the publishing house Vrin – which requested a work on history of philosophy and not one on philosophy of action – he had to delete and revise various paragraphs or expressions from his PhD thesis explicitly referring to the topic of action. For instance, the label “school of activity”, which is central to his PhD thesis, was replaced by “reflexive school” in the published version. Without meaning to undermine the theoretical value of the published version, I believe that in order to fully understand the fundamental role that Roth attributes to philosophy of action in Canguilhem, it is far more pertinent to refer to his PhD thesis. Therefore, this is the version I will be mainly quoting further on.

5 As can be read in the *Critique of Pure Reason*, see (Kant, 1998, p. 210, A77/B103; p. 245, B129).

6 The teacher who had taught him the precepts of Kant’s doctrine (see Sect. 2).

7 See (Canguilhem, 2018, p. 1351). Here I refer to a debate recorded and broadcasted on French television on the 27th of February 1968. The transcript of the debate was then published in *Revue de l’enseignement philosophique* 18/2, December 1967/January 1968, 65–72. François Dagognet (1924–2015) was a French philosopher and pupil of Canguilhem. Among his works on philosophy of biology were *Philosophie biologique* (1962) and *Le vivant* (1988). He was also the author of *Georges Canguilhem. Philosophe de la vie* (1997).

8 Organism as a concept: Canguilhem well understood, as Debru (1980, p. 487) wrote, that the term “organism” does not appear in the third *Critique* but only later, in *Opus Postumum*.

9 See (Debru, 2018, p. 302).

10 See Sect. 3.

11 Without counting a brief review of a book by Pierre-Maxime Schuhl, *Machinisme et philosophie*. However, even the latter made use of certain references to the subject of technique: machinery was defined as a “technical phenomenon”, see (Canguilhem, 2011, p. 511).

12 On some uses of the concept of practice in the first *Critique*, see for example (Guyer, 1989).

13 Jules Lagneau (1851–1894) was a French philosopher who taught in several high schools in France. Parts of his philosophy lessons were collected posthumously in *Célèbres leçons et fragments* (1950) and *Jules Lagneau, cours intégral 1886–87* (1996).

14 Jules Lachelier (1832–1918) was a French philosopher. His most famous work, in which one can detect the influence of Kant’s philosophy, is *Du fondement de l’induction* (1871).

15 See Canguilhem (2018, pp. 1124–1125).

16 Now reprinted in Canguilhem (2011, pp. 935–978).

17 *Libres Propos (Journal d’Alain)* was the title of a journal launched in 1921 by Jeanne Alexandre and her husband Michel Alexandre, a follower of Alain.

18 Particularly Georges Bénézé (1888–1978), another of Alain’s students. Canguilhem critiques Bénézé’s article published in the *Libres propos* on 20 October 1929.

19 See (Kant, 1998, p. 273 – KrV, A140/B181).

20 Camille Planet (1892–1963) was a French professor of philosophy. The *Traité* was the only book he published.

21 Canguilhem quotes H. Bergson, *La pensée et le mouvant*, 124, online edition: http://classiques.uqac.ca.

22 Canguilhem quotes the French philosopher René Le Senne (1882–1954), who defined the “metaphysical” as a “professor of unity” in his book *Le devoir*, Paris, Alcan, 1930, 384.

23 On Canguilhem and technique, see (Sebestik, 1993).

24 Canguilhem here refers to the edition of the *Rules* edited by C. Adam and P. Tannery, which I will also cite in what follows.

25 On the notion of failure and error in Canguilhem’s conception of technique, see (Talcott, 2019).

26 For the difference between analysis and synthesis, see Kant (1998, p. 130 – KrV, B10/A8).

27 Petrus Camper dealt mainly with anatomy, physiology, palaeontology, zoology, and physics. He wrote several books, including one entitled *On the Best Form of Shoe*, (1861) originally published as *Verhandeling over den besten schoen* (1781).

28 Silvestro Marcucci (1931–2005), who founded the scientific journal *Studi Kantiani* in 1988, directed Marianetti’s dissertation, entitled *La riflessione storica e teorica di Georges Canguilhem sulla biologia e la medicina* (University of Pisa, 1992), available at the CAPHÉS, GC. 2. 8. The reception of Canguilhem’s work in Italy (which began in 1970 with a Mauro di Giandomenico’s review of the *Études d’histoire et de philosophie des sciences* and continued with studies by authors such as Giuseppe Quarta and Giuseppe Sertoli up to the recent work by Michele Cammelli (see Cammelli, 2022) is a broad and varied topic to which I dedicated a specific article, see (Sfara, 2014).

29 Which can be consulted at the CAPHÉS, GC. 16. 1.

30 Paul Langevin (1872–1946) was a French physicist with Communist-inspired political ideas. His books include *La pensée et l’action* (1950).

31 Paul Rivet (1876–1958) was a French ethnologist with political ideas inspired by socialism.

32 These are the words of Canguilhem, as reported by Cammelli (2011, p. 519).

33 Among non-philosophical disciplines that have been used by Canguilhem for his philosophical reflection, we may quote, in addition to medicine, geography: see (Talcott, 2019, p. 63–68). I have discussed Canguilhem’s topic of technique in Sfara (2016, 2018). For further insights on this topic, see also Méthot (2013) and Sebestik (1993).

34 See (Alain, 1920, p. 75).

35 Francis Courtès, *maître-assistant* at the Montpellier Faculty of Letters and Social Sciences, see (Canguilhem, 1991, p. 295).

36 Kant, Anth, AA 15: 964. 06–09.

37 Canguilhem (1991, p. 171) quotes here an article by Pales and Monglond: “Le taux de la glycémie chez les noirs en A. E. F. et ses variations avec les états pathologiques”, *La presse médicale*, 13/05/1934, p. 767.

38 This is one of the three letters that form the correspondence between Marianetti and Canguilhem, which took place between January and February 1993 (CAPHÉS, GC. 2.8.2). Marianetti wrote the first letter to Canguilhem to inform him that he would be writing an article about his philosophy (which is the article we quoted above, see Marianetti, 2013). He took the opportunity to ask Canguilhem the following question: “Can your notion of normativity agree with the Kantian notion of purpose?”. Hence Canguilhem’s answer above.

39 On the Kantian notion of “concept”, which in the transcendental subject allows to avoid the dispersion of a disorderly multiplicity of empiric data in line with the first *Critique*, see (Cutro, 2011, p. 13). Just like Cutro, I am also convinced that such an interpretation, linked to the first *Critique*, exists also in Canguilhem. However, my argument is that another side of this interpretation is at work in Canguilhem’s philosophy – which, as explained above, is related to the notion of “concept” that we find in texts such as *La formation du concept de réflexe*. In my opinion, if we refer to Kant, this is a notion that stems from Kant’s third *Critique.*

40 The volume in question, part of Canguilhem’s personal library, is now collected at the CAPHÉS, location CAN 730. In particular, Canguilhem pencilled the title of the already quoted work by Camper (see Sect. 4) and added “on Camper, see Jurgis Baltrusaitis, *Aberrations”*. This work by Baltrusaitis, entitled *Aberrations: quatre essais sur la légende des formes*, was published for the first time in 1957, which leaves no doubt as to the fact that the notes we are discussing could not have been written prior to that date. I thank Camille Limoges for this information.

41 This text does not have an exact date. However, because of its location at the CAPHÉS, it almost certainly dates back to the 1920s. Moreover, it is not clear whether it contains notes directly taken by the young Canguilhem during Alain’s classes or it merely presents Kant’s philosophy from the young Canguilhem’s point of view (in any case, with a visible influence of Alain’s interpretation). Anyhow, there is also another manuscript at the CAPHÉS that brings together a series of notes taken during Alain’s lessons on Kant (as it was unambiguously indicated by Canguilhem on the first page) which can be found in the location GC. 3.3.3.

42 This is the text of a course that Canguilhem held at the Sorbonne in the academic year 1966–67.

## References

[CR1] Alain (1893). Dialogue philosophique entre eudoxe et ariste. Revue de Métaphysique et de Morale.

[CR2] Alain (1896). Quatrième dialogue philosophique entre eudoxe et ariste. Revue de Métaphysique et de Morale.

[CR3] Alain. (1900). Le problème de la perception. *Revue de Métaphysique et de Morale*, 8, 745–754. Retrieved from http://classiques.uqac.ca/classiques/Alain/probleme_de_la_perception/probleme_de_la_perception.html

[CR4] Alain. (1902). L’idée d’objet. *Revue de Métaphysique et de Morale*, 10, 409–421. Retrieved from http://classiques.uqac.ca/classiques/Alain/idee_objet/idee_objet.html

[CR5] Alain. (1920). *Système des beaux–arts*. Gallimard. Retrieved from http://classiques.uqac.ca/classiques/Alain/Alain.html

[CR6] Alain. (1931). *Vingt leçons sur les beaux–arts*. Gallimard. Retrieved from http://classiques.uqac.ca/classiques/Alain/Alain.html

[CR7] Alain. (1936). *Histoire de mes pensées*. Gallimard. Retrieved from http://classiques.uqac.ca/classiques/Alain/Alain.html

[CR8] Alain. (1939). *Idées*. Hartmann. Retrieved from http://classiques.uqac.ca/classiques/Alain/Alain.html

[CR9] Alain. (1940). *Éléments de philosophie*. Gallimard. Retrieved from http://classiques.uqac.ca/classiques/Alain/Alain.html

[CR10] Alain. (1946). *Lettres à Sergio Solmi sur la philosophie de Kant*. Hartmann. Retrieved from http://classiques.uqac.ca/classiques/Alain/lettres_sergio_solmi_Kant/lettres_solmi_Kant.html

[CR11] Bateson P (2005). The return of the whole organism. Journal of Biosciences.

[CR12] Beaufret, J. (1984). *Notes sur la philosophie en France au 19ème siècle*. Vrin.

[CR13] Bing, F., & Braunstein, J. F. (2018). Entretien avec Georges Canguilhem. In G. Canguilhem (Ed.), *Œuvres complètes*, *vol. V. Histoire des sciences, épistémologie. Commémorations*, *1966–1995* (pp. 1281–1300). Vrin.

[CR14] Braunstein JF (2000). Canguilhem avant Canguilhem. Revue d’histoire des sciences.

[CR15] Brilman M (2018). Canguilhem’s critique of Kant: Bringing rationality back to life. Theory Culture & Society.

[CR16] Brunschvicg, L. (1954). *Écrits philosophiques II. L’orientation du rationalisme*. PUF. Retrieved from http://classiques.uqac.ca/classiques/brunschvicg_leon/brunschvicg_leon.html.

[CR17] Cammelli, M. (2011). Présentation – Le fascisme et les paysans. In G. Canguilhem (Ed.), *Œuvres complètes, vol. I. Écrits philosophiques et politiques, 1926–1939* (pp. 515–533). Vrin.

[CR18] Cammelli, M. (2022). *Canguilhem philosophe. Le sujet et l’erreur*. PUF.

[CR19] Canguilhem, G. (1924). *Kant, cours de chartier*. Unpublished work, CAPHÉS, GC. 3. 3. 3.

[CR20] Canguilhem, G. (1929). À la gloire d’Hippocrate, père du tempérament. *Libres propos (Journal d’Alain)*, 20 août 1929. In G. Canguilhem (Ed.), *Œuvres complètes, vol. I. Écrits philosophiques et politiques, 1926–1939* (pp. 248–250). Vrin.

[CR21] Canguilhem, G. (1929-32). *Philosophie (éléments de doctrine et textes choisis)*. Unpublished work, CAPHÉS, GC. 8.

[CR22] Canguilhem, G. (1931-32). *La création continuée*. Unpublished work, CAPHÉS, GC. 12. 1. 16.

[CR23] Canguilhem, G. (1941-42). *Cours donné pour la Faculté des Lettres de Strasbourg à Clermont Ferrand*. Unpublished work, CAPHÉS, GC. 11. 1.

[CR24] Canguilhem, G. (1943). *Essai sur quelques problèmes concernant le normal et le pathologique*. Imprimerie la Montagne.

[CR25] Canguilhem, G. (1947-48). *Le problème de la création*. Unpublished work, CAPHÉS, GC. 12. 1.

[CR26] Canguilhem, G. (1955). *La formation du concept de réflexe aux XVIIe et XVIIIe siècles*. PUF.

[CR27] Canguilhem, G. (1962-63). *Science et technique, I.H.S* Unpublished work, CAPHÉS, GC. 16. 1.

[CR28] Canguilhem, G. (2008). Machine and organism. In G. Canguilhem (Ed.), *Knowledge of life* (pp. 75–97). Fordham University Press.

[CR29] Canguilhem, G. (2008b). Réflexions sur la création artistique selon Alain, *Cahiers Philosophiques*, hors série, septembre, 55–70.

[CR30] Canguilhem, G. (1991). *The normal and the pathological*. Zone Books.

[CR31] Canguilhem, G. (2011). *Œuvres complètes, vol. I. Écrits philosophiques et politiques, 1926–1939*. Vrin.

[CR32] Canguilhem, G. (2015). *Œuvres complètes, vol. IV. Résistance, philosophie biologique et histoire des sciences, 1940–1965*. Vrin.

[CR33] Canguilhem, G. (2018). *Œuvres complètes*, *vol. V. Histoire des sciences, épistémologie. Commémorations 1966–1995*. Vrin.

[CR34] Canguilhem, G. (2019). *Œuvres complètes, vol. III. Écrits d’histoire des sciences et d’épistémologie*. Vrin.

[CR35] Canguilhem, G. (undated) (Ed.). *Kant*. Unpublished work, CAPHÉS, GC. 4. 7.

[CR36] Cutro, A. (2011). *Technique et vie. Biopolitique et philosophie du bios dans la pensée de Michel Foucault*. L’Harmattan.

[CR37] Debru C (1980). L’introduction du concept d’organisme dans la philosophie kantienne: 1790–1803. Archives de Philosophie.

[CR38] Debru C (2018). Philosophie et biologie: La Connaissance de la vie et les enseignements de Canguilhem à la faculté des lettres de Strasbourg (1941–1948). Revue d’Histoire des Sciences.

[CR39] Descartes, R. (2006). *A discourse on the method of correctly conducting one’s reason and seeking truth in the sciences*. Oxford University Press.

[CR40] Descartes, R. (1963). *Œuvres philosophiques de Descartes* (I vol.). Garnier Frères.

[CR41] Descartes, R. (1985). *The philosophical writings of Descartes* (Vol. I). Cambridge University Press.

[CR42] Desmond, H., & Huneman, P. (2020). The ontology of organismic agency: A Kantian approach. In A. Altobrando & P. Biasetti (Eds.), *Natural born monads: On the metaphysics of organisms and human individuals* (pp. 33–64). De Gruyter. 10.1515/9783110604665-003

[CR43] Etxeberria A, Wolfe CT (2018). Canguilhem and the logic of life. Transversal: International Journal for the Historiography of Science.

[CR44] Fedi, L. (2001). L’esprit en marche contre les codes: Philosophie des sciences et dépassement du kantisme chez Léon Brunschvicg. In L. Fedi, & J. M. Salanskis (Eds.), *Les philosophies françaises et la science: Dialogue avec Kant* (pp. 119–142). ENS éditions.

[CR45] Fedi, L. (2018). *Kant, une passion française, 1795–1940*. Verlag.

[CR46] Foucault, M. (1991). Introduction. In G. Canguilhem (Ed.), *The normal and the pathological* (pp. 7–24). Zone Books.

[CR47] Gayon, J. (2006). Le concept d’individualité dans la philosophie biologique de Georges Canguilhem. In M. Bitbol & J. Gayon (Eds.), *L’épistémologie française, 1830–1970* (pp. 389–419). Éditions Matériologiques.

[CR48] Gayon, J., & Petit, D. (2018). *La connaissance de la vie aujourd’hui*. ISTE editions.

[CR49] Goy, I., & Watkins, E. (Eds.). (2014). *Kant’s theory of biology*. De Gruyter.

[CR50] Guillin V (2008). Les études cartésiennes de Georges Canguilhem. Les Cahiers philosophiques.

[CR51] Guillin V (2015). Descartes à travers mes âges”. Retour sur quelques lectures cartésiennes de Canguilhem. Revue de Métaphysique et de Morale.

[CR52] Guyer P (1989). The unity of reason: Pure reason as practical reason in Kant’s early conception of the transcendental dialectic. The Monist.

[CR53] Huneman, P. (2008). *Métaphysique et biologie. Kant et la constitution du concept d’organisme*. Kimé.

[CR54] Kant, I. (1974). *On the old saw: That may be right in theory but it won’t work in practice*. University of Pennsylvania Press.

[CR55] Kant, I. (1998). *Critique of pure reason. The Cambridge edition of the works of Immanuel Kant*. Cambridge University Press.

[CR56] Kant, I. (2000). *Critique of the power of judgment. The Cambridge edition of the works of Immanuel Kant*. Cambridge University Press.

[CR57] Lachelier, J. (1896). *Du fondement de l’induction. Suivi de psychologie et métaphysique*. Alcan.

[CR58] Lagneau, J. (1964). *Célèbres leçons et fragments*. PUF. Retrieved from http://classiques.uqac.ca/classiques/lagneau_jules/celebres_lecons_et_fragments/celebres_lecons.html.

[CR59] Lefève C (2014). De la philosophie de la médecine de Georges Canguilhem à la philosophie du soin médical. Revue de Métaphysique et de Morale.

[CR60] Limoges, C. (1994). Critical bibliography. In F. Delaporte (Ed.), *A vital rationalist. Selected writings from Georges Canguilhem* (pp. 385–454). Zone Books.

[CR61] Limoges, C. (2012). L’épistémologie historique dans l’itinéraire intellectuel de Georges Canguilhem. In *Epistemology and history from Bachelard and Canguilhem to today’s history of science* (pp. 53–66). Max Planck Institute for the History of Science.

[CR62] Limoges, C. (2015). Introduction. In G. Canguilhem (Ed.), *Œuvres complètes, vol. IV. Résistance, philosophie biologique et histoire des sciences, 1940–1965* (pp.7–48). Vrin.

[CR63] Limoges, C. (2018). Introduction. In G. Canguilhem (Ed.), *Œuvres complètes*, *vol. V. Histoire des sciences, épistémologie. Commémorations*, *1966–1995* (pp. 7–57). Vrin.

[CR64] Lupi, F. (2019). Tra Canguilhem e Alain. Normatività, immaginazione e creazione tecnica. In F. Lupi & S. Pilotto (Eds.), *Infrangere le norme. Vita, scienza e tecnica nel pensiero di Georges Canguilhem* (pp. 119–149). Mimesis.

[CR65] Marianetti M (1994). Canguilhem, Kant e la filosofia trascendentale. Studi Kantiani.

[CR66] McLaughlin, P. (1990). *Kant’s critique of teleology in biological explanation, antinomy and teleology*. E. Meller Press.

[CR67] Méthot PO (2013). On the genealogy of concepts and experimental practices: Rethinking Georges Canguilhem’s historical epistemology. Studies in History and Philosophy of Science.

[CR68] Méthot, P. O., & Sholl, J. (Eds.). (2020). *Vital norms: Canguilhem’s the normal and the pathological in the twenty-first century*. Hermann.

[CR69] Moreno, A., & Mossio, M. (2015). *Biological autonomy. A philosophical and theoretical enquiry*. Springer.

[CR70] Nicholson DJ (2014). The return of the organism as a fundamental explanatory concept in biology. Philosophy Compass.

[CR71] Rand S (2011). Organism, normativity, plasticity: Kant, Canguilhem, Malabou. Continental Philosophy Review.

[CR72] Roth, X. (2010). *Georges Canguilhem et l’école française de l’activité. Juger et agir (1926–1939)*. PhD thesis. Université d’Aix-Marseille. CAPHÉS, (H) Cang 41.

[CR73] Roth, X. (2011). Présentation – Traité de logique et de morale. In G. Canguilhem (Ed.), *Œuvres complètes, vol. I. Écrits philosophiques et politiques, 1926–1939* (pp. 597–630). Vrin.

[CR74] Roth, X. (2013). *Georges Canguilhem et l’unité de l’expérience. Juger et agir, 1926–1939*. Vrin.

[CR75] Salomon-Bayet C (1996). Georges Canguilhem, le concept et l’action. Raison Présente.

[CR76] Sánchez Madrid, N. (2017). La réflexion, le concept et la vie. La présence de Georges Canguilhem dans l’épistémologie critique de Gérard Lebrun. In M. Cohen-Halimi, V. de Figueiredo & N. Sánchez Madrid (Eds.), *Gérard Lebrun philosophe* (pp. 137–156). Beauchesne.

[CR77] Schmidgen H (2014). The life of concepts: Georges Canguilhem and the history of science. History and Philosophy of the Life Sciences.

[CR78] Sebestik, J. (1993). Le rôle de la technique dans l’œuvre de Georges Canguilhem. In É. Balibar, et al. (Eds.), *Georges Canguilhem, philosophe, historien des sciences* (pp. 243–250). Albin Michel.

[CR79] Sholl, J. (2020). Plastic, variable, and constructive: Renewing Canguilhem biological normativity. In P. O. Méthot, & J. Sholl (Eds.), *Vital norms: Canguilhem’s the normal and the pathological in the twenty-first century* (pp. 255–293). Hermann.

[CR80] Talcott, S. (2019). *Georges Canguilhem and the problem of error*. Palgrave MacMillan.

[CR81] Walsh, D. M. (2015). *Organisms, agency, and evolution*. Cambridge University Press.

[CR82] Zammito, J. H. (1992). *The genesis of Kant’s critique of judgement*. University of Chicago Press.

[CR83] Zumbach, C. (1984). *The transcendent science. Kant’s conception of biological methodology*. Nijhoff.

[CR84] Sfara, E. (2016). *Una filosofia della prassi. Organismi, arte e visione in Georges Canguilhem*. Nuova Trauben.

[CR85] Sfara, E. (2018). *Georges Canguilhem inédit. Essai sur une philosophie de l’action*. L’Harmattan.

[CR86] Sfara, E. (2014). Georges Canguilhem filosofo tout court: spunti e riflessioni sulla ricezione italiana delle sue opere. In I. Pozzoni (Ed.), *Schegge di filosofia XI* (pp. 125–137). Decomporre Edizioni.

[CR87] Schwartz, Y. (2011). Jeunesse d’un philosophe. In G. Canguilhem (Ed.), *Œuvres complètes, vol. I. Écrits philosophiques et politiques, 1926–1939* (pp. 71–99). Vrin.

[CR88] Piquemal, J. (1985). G. Canguilhem, professeur de Terminale: un essai de témoignage. *Revue de Métaphysique et de Morale**90*(1), 63–83.

