# Correction to: From technique to normativity: the influence of Kant on Georges Canguilhem’s philosophy of life

**DOI:** 10.1007/s40656-023-00589-0

**Published:** 2023-06-21

**Authors:** Emiliano Sfara

**Affiliations:** Institute of Biology, National Institute in Science and Technology in Interdisciplinary and Transdisciplinary Studies in Ecology and Evolution (INCT-IN-TREE), Federal University of Bahia, Bahia, Brazil

History and Philosophy of the Life Sciences (2023) 45:16

10.1007/s40656-023-00573-8

The article “From technique to normativity: the influence of Kant on Georges Canguilhem’s philosophy of life”, written Emiliano Sfara, was originally published Online without Open Access. After publication in volume 45, issue 2, Article 16 the author decided to opt for Open Choice and to make the article an Open Access publication. Therefore, the copyright of the article has been changed to ‘The Author(s) 2023’ and the article is forthwith distributed under the terms of the Creative Commons Attribution. This article is licensed under a Creative Commons Attribution 4.0 International License, which permits use, sharing, adaptation, distribution and reproduction in any medium or format, as long as you give appropriate credit to the original author(s) and the source, provide a link to the Creative Commons licence, and indicate if changes were made. The images or other third party material in this article are included in the article’s Creative Commons licence, unless indicated otherwise in a credit line to the material. If material is not included in the article’s Creative Commons licence and your intended use is not permitted by statutory regulation or exceeds the permitted use, you will need to obtain permission directly from the copyright holder. To view a copy of this licence, visit http://creativecommons.org/licenses/by/4.0/.

